# Angiomyolipoma and Malignant PEComa: Discussion of Two Rare Adrenal Tumors

**DOI:** 10.1155/2016/5204092

**Published:** 2016-02-22

**Authors:** Douglas Kwazneski II, Megan Merrill, Jessica Young, Harry Sell

**Affiliations:** ^1^University of Florida, Gainesville, FL 32608, USA; ^2^The Ohio State University, Columbus, OH 43210, USA; ^3^Roswell Park Cancer Institute, Buffalo, NY 14263, USA; ^4^UPMC Mercy, Pittsburgh, PA 15219, USA

## Abstract

Angiomyolipoma and PEComa are rare tumors descending from perivascular epithelial cells (PECs), with distinctive IHC, morphological, and ultrastructural features. The kidney is the most frequent site of origin, but not the only one; however, adrenal gland angiomyolipomas are extremely rare. We describe two cases being found in the adrenal glands. Given the paucity of literature on the subject, more information on this disease is necessary for diagnosis and treatment. Here, we describe two complete case reports, from presentation to treatment and follow-up, along with imaging and microscopic pathology samples, and provide a comprehensive review as to the history and current literature available regarding these extremely rare tumors.

## 1. Introduction

Angiomyolipomas are mesenchymal tumors made up of abnormal blood vessels, spindle cells, and mature adipocytes, believed to be derived from perivascular epithelioid cells (PEC) [1]. PEComas are a family of mesenchymal tumors arising from PECs, including angiomyolipomas, as well as pulmonary lymphangioleiomyomatosis [2, 3]. We recently removed a large right adrenal tumor with multiple calcifications suspicious for adrenal carcinoma on imaging. Pathology, however, indicated angiomyolipoma (AML), only the fourth AML of the adrenal gland in the English literature. Another 11 cm right adrenal tumor we resected had suspicious elements. An independent review indicated that this tumor was a malignant PEComa. It is the first PEComa of the adrenal gland we and the independent reviewer have seen and is one of the first, to our knowledge, to be reported in the English literature.

## 2. Case Reports

### 2.1. Case  1

65 y/o Caucasian female with hypothyroidism and coronary artery disease presented with intermittent right upper quadrant abdominal pain for one year, worsening over a month. She denied fevers and weight changes, was tolerating her diet without difficulty, and had noticed no change in bowel movements. She reported no history of nephrolithiasis, hematuria, or dysuria. Physical exam was unremarkable except for mild right flank tenderness.

**Figure 1:**
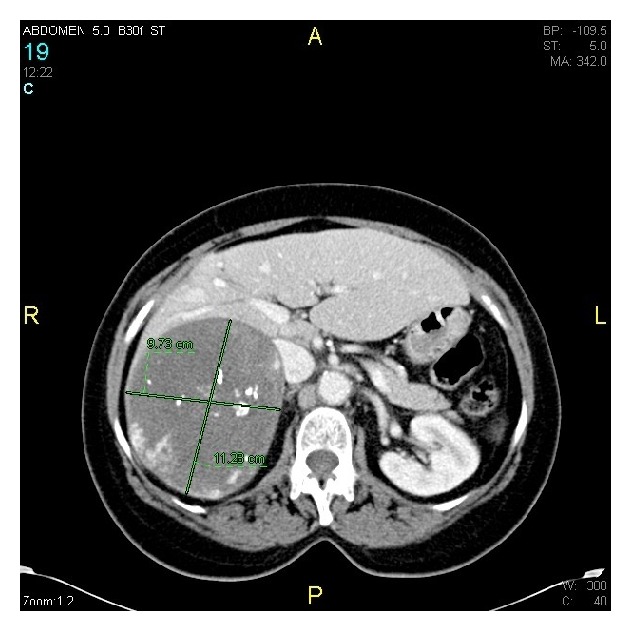
CT scan showing 11.3 cm × 9.7 cm right adrenal mass with scattered calcifications.

Abdominal U/S revealed a large heterogeneous mass, appearing to arise from the right adrenal gland. CT scan confirmed an 11.3 × 9.4 cm right adrenal mass with scattered calcifications consistent with adrenal CA (Figure 1). 24-hour urinary catecholamines, metanephrines, plasma cortisol, and aldosterone were normal.

**Figure 2:**
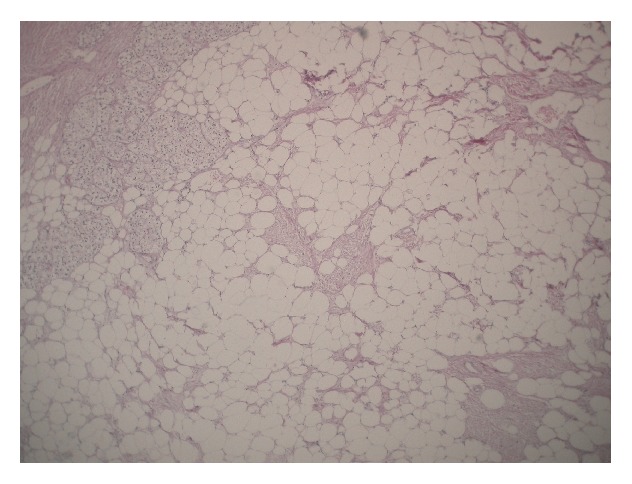
Thick-walled blood vessels and smooth muscle spindle cells interspersed among numerous adipocytes (magnification 200x, hematoxylin and eosin stain).

Given the suspicion for adrenal CA, the patient underwent an open right adrenalectomy. The mass displaced surrounding structures with no evidence of invasion. Intraoperative frozen sections demonstrated necrotic tissue without signs of malignancy. The 626 g, 13 × 12 × 9 cm specimen contained adipose tissue, thick walled blood vessels, and scattered foci of spindle cells with areas of hemorrhage and calcifications on H&E stain (Figure 2). Immunohistochemistry was consistent with angiomyolipoma, with positive smooth muscle actin on the spindle cells. The final diagnosis was adrenal angiomyolipoma. The patient was discharged on POD #5 without complications and as of 36-month follow-up had no evidence of disease.

### 2.2. Case  2

**Figure 3:**
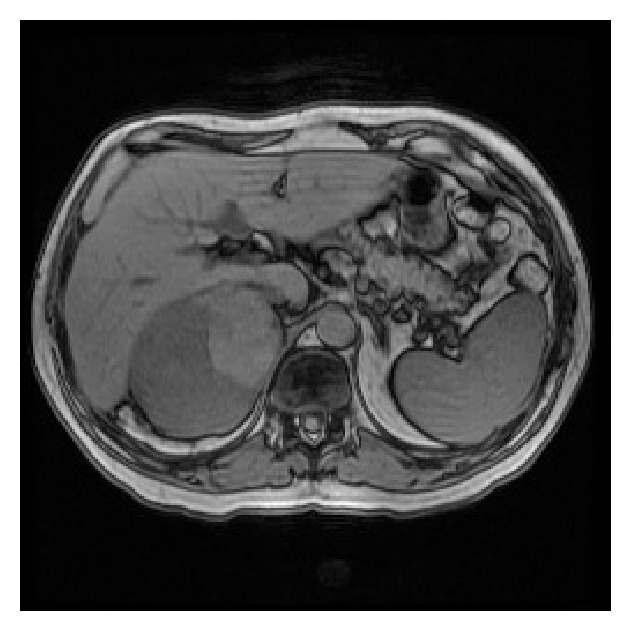
Complex right adrenal mass on MRI with no evidence of invasion.

**Figure 4:**
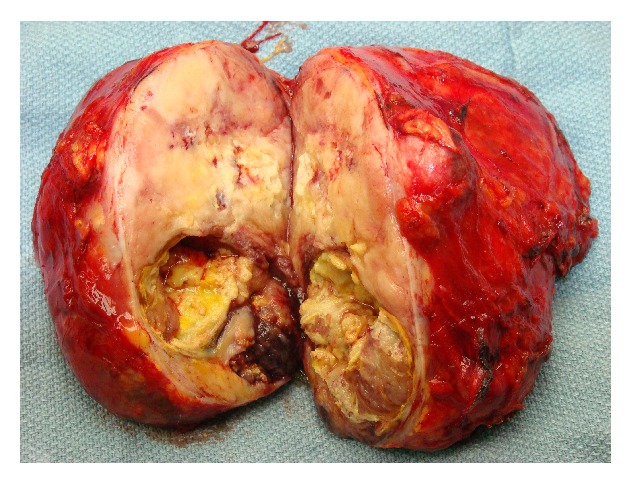
Right adrenal PEComa. It was a 14.5 × 12 × 7 cm fleshy tumor with necrosis and hemorrhage.

63 y/o Caucasian male without past medical history presented with chronic cough, for which a CT scan of the sinuses and chest was performed. The lower images of the CT showed a possible right kidney lesion. CT of the abdomen and pelvis demonstrated a 13 cm right adrenal mass, consistent with adrenal CA. MRI clarified the lesion as adrenal with no invasion of surrounding structures (Figure 3). The patient underwent a right open adrenalectomy. The mass showed no involvement of surrounding structures but did compress the IVC. The mass was excised and sent to pathology (Figure 4).

**Figure 5:**
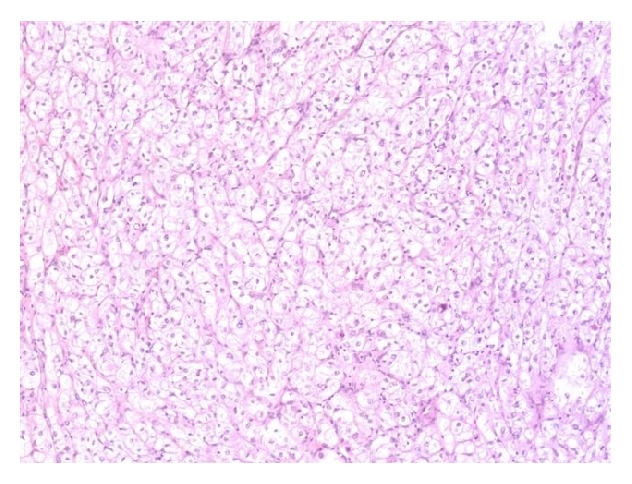
Focal alveolar pattern. These areas are suggestive of an alveolar soft part sarcoma.

**Figure 6:**
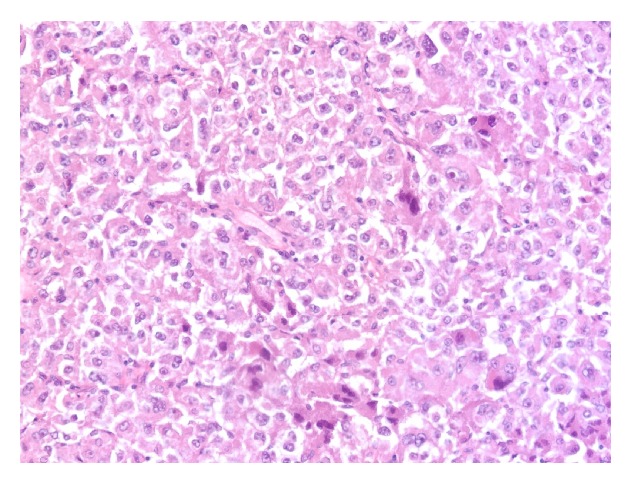
PAS stains carbohydrate moieties such as glycogen. This was a granular material more consistent with a PEComa.

The tumor replaced the adrenal gland in its entirety but the capsule was intact, with various growth patterns (Figures 5 and 6). Histology showed a focal alveolar pattern and PECs. Immunohistochemistry was positive for SMA, desmin, and MITF, but negative for HMB-45, TFE3, and Pan-Keratin; our pathologists felt this was consistent with PEComa of the right adrenal gland. Due to the size (13 cm), the infiltrative growth pattern, and the presence of necrosis, the tumor was classified as malignant as per the Folpe criteria [4]. The patient was discharged on POD #3 and underwent 3-month follow-ups for 2 years followed by 6-month follow-ups with imaging. He did not receive chemotherapy or radiation. Presently, he has no evidence of disease.

## 3. Discussion

In 2002, the World Health Organization (WHO) recognized a family of neoplasms showing morphologic and immunohistochemical evidence of perivascular epithelioid cell (PEC) differentiation, including angiomyolipoma (AML), lymphangioleiomyomatosis (LAM), and other clear cell tumors [2]. The perivascular epithelial cell is consistently present. It has distinctive IHC and morphologic and ultrastructural features, such as an epithelioid appearance with clear or granulated cytoplasm and a round to oval centrally located nucleus. This is one of the first reported cases of a PEComa in the adrenal gland and only the fourth reported case in the English literature of an adrenal angiomyolipoma.

### 3.1. Angiomyolipoma

The term “angiomyolipoma” was first coined in 1951 by Morgan to describe a benign renal tumor composed of blood vessels, muscle, and fat [5]. Thick-walled blood vessels that lack elastic fibers found in normal arteries are characteristic, making angiomyolipomas prone to spontaneous rupture and retroperitoneal hemorrhage. One institution notes that seven cases of spontaneous retroperitoneal hemorrhage over ten years were actually ruptured angiomyolipomas [6, 7]. The smooth muscle portion of the tumor is comprised of spindle cells and epithelioid cells. The nuclei are usually small, without mitotic activity; however, mitotic activity has been documented and should not be confused with sarcoma. Large, mature fat cells comprise the remainder, similar to other lipomatous tumors.

The diagnosis of angiomyolipoma is made by the presence of components listed above, as well as using immunohistochemistry. In most cases, the tumors stain positive for desmin, muscle specific actin, and HMB45. Markers for epithelial cells, cytokeratins, and epithelial membrane antigen are generally absent [7].

Angiomyolipoma is the most common benign resectable tumor of the kidney and is rare in other locations. As few as 40 documented cases of extrarenal angiomyolipoma exist and only three cases of adrenal angiomyolipoma have been reported. The first two cases of adrenal angiomyolipoma were published in 2001 in the Journal of Clinical Pathology by Lam and Lo. They described a 46 y/o female incidentally found to have an 8 cm left adrenal mass. The second case was a 20 y/o male with tuberous sclerosis who noted hematuria and left groin pain. He was found to have multiple angiomyolipomas in both kidneys, the liver, and the left adrenal gland [7]. In 2005, Elsayes documented a third case of adrenal angiomyolipoma in a 49 y/o female with tuberous sclerosis found to have a large right adrenal mass on MRI, diagnosed as adrenal angiomyolipoma after surgical excision [8]. Our patient, the fourth documented case in English literature, presented with right upper quadrant pain. Initially, her diagnosis was suspicious for adrenocortical carcinoma due to calcifications noted on CT scan.

Imaging of angiomyolipomas demonstrates characteristic aneurysmal dilation, representing thick-walled vessels [8, 9]. It can be difficult to distinguish angiomyolipomas from other tumors sharing fat densities. In most cases, angiomyolipomas are an incidental finding on imaging studies [10]. Close follow-up is necessary, given the inability to distinguish these benign lesions from malignancy. AML has been considered a hamartoma in the past, but presently its clonal nature has been demonstrated [11–13]. It is associated with tuberous sclerosis and the 3rd to 4th decade of life and is most frequent in patients of female sex [14].

### 3.2. PEComa

The first perivascular epithelioid cell (PEC) was noted by Apitz in 1943 in renal angiomyolipomas. The term PEComa was introduced in 1996 by Zamboni to describe a tumor arising in the pancreas [15]. Since then, the tumors have been noted in many organs, though ours is one of the first known in the adrenal gland. Most PECs express myogenic and melanocytic markers like HMB-45, HMSA-1, MelanA/mart1, microphthalmia transcription factor (MITF) actin, and desmin [3]. Our malignant PEComa was positive for SMA, desmin, and MITF, but negative for HMB-45, TFE3, and Pan-Keratin. The tumor being negative for HMB-45 is rare, as nearly all are positive for HMB-45 [4], but PEComas being negative for HMB-45 have been reported elsewhere [16]. A hypothesized explanation for the variance in HMB-45 expression could be related to the tuberous sclerosis complex (which our patient had), in which there are defects in the enzymes involved in the conversion of phenylalanine to melanin or catecholamines [16].

As with angiomyolipomas, with PEComas, there is an association with tuberous sclerosis, an autosomal dominant syndrome. This rare, multisystem genetic disease results from mutations from one of two genes, TSC1 or TSC2. These genes encode tumor growth suppressor proteins hamartin and tuberin, causing tumors to grow in the brain, kidney, heart, lungs, eyes, and skin. Historically, neurological issues were the primary cause of death for these patients, but as medical care has progressed, renal disease has become the primary cause of death. Given that these patients develop masses at multiple sites, including the kidneys, it was observed that these patients develop renal cysts and masses in childhood, and the incidence only increases with age. 60–80% of these patients will have angiomyolipomas, of which PEComas are a modulated cell type [17].

### 3.3. Epidemiology

There is a marked increase in incidence in patients of female sex (6-7 : 1), and age at diagnosis ranges within 8–89 years. There is a high level of association (60–80%) with tuberous sclerosis. The most frequent sites of PEComas are the uterus and retroperitoneum, though the tumor can be found virtually anywhere [17–24]. The patient often presents with focal pain, but these tumors are also incidentally found on imaging [10]. Neither clinical presentation nor radiologic appearance allows preoperative diagnosis.

### 3.4. Malignant PEComas

**Figure 7:**
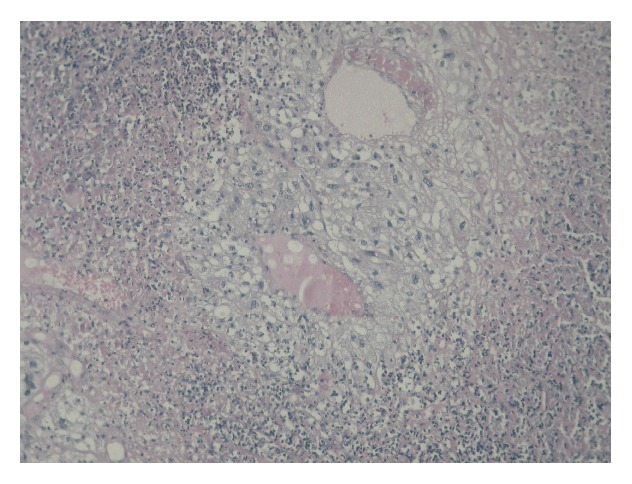
Tumor necrosis.

A small subset of PEComas show aggressive behavior and can be malignant. Folpe described high-risk criteria for malignancy, including size > 5 cm, mitotic rate > 1/50 hpf, necrosis, high nuclear grade and cellularity, vascular invasion, and an infiltrative growth pattern [4] (Figure 7). If a tumor has none of these, it is considered benign. If the tumor meets less than two criteria, malignancy is uncertain. If there are two or more high-risk features, such as in our case, then that is classified as malignant. These criteria are not well-established, however, as this tumor is rare and follow-up has not been consistent. A follow-up review by Folpe in 2012 reevaluated the previous criteria with new data and found that the rate of “aggressive behavior” in tumors classified as malignant was 51%, less than the original 71% proposed. This led to a revised set of Folpe criteria, consisting only of size > 5 cm and high mitotic rate [25]. A few malignant PEComas have metastasized after several years [26–28]. Complete surgical resection has been the treatment of choice, as the role of chemotherapy and radiation is unknown.

## 4. Conclusion

This is one of the first noted cases of an adrenal malignant PEComa, and the fourth adrenal angiomyolipoma. These tumors appear to represent a spectrum of diseases derived from the perivascular epithelioid cell (PEC). Both of these patients have been followed up for over 36 months without evidence of recurrence or metastatic spread from the malignant PEComa. As more of these tumors are described, open questions will likely be answered.

## References

[1] Flum A. S., Hamoui N., Said M. A. (2016). Update on the diagnosis and management of renal angiomyolipoma. *The Journal of Urology*.

[2] Folpe A. L., Fletcher C. D. M., Unni K. K., Epstein J., Mertens F. (2002). Neoplasms with perivascular epithelioid cell differentiation (PEComas). *Pathology and Genetics of Tumours of Soft Tissue and Bone*.

[3] Martignoni G., Pea M., Reghellin D., Zamboni G., Bonetti F. (2008). PEComas: the past, the present and the future. *Virchows Archiv*.

[4] Folpe A. L., Mentzel T., Lehr H.-A., Fisher C., Balzer B. L., Weiss S. W. (2005). Perivascular epithelioid cell neoplasms of soft tissue and gynecologic origin: a clinicopathologic study of 26 cases and review of the literature. *American Journal of Surgical Pathology*.

[5] Morgan G. S., Straumfjord J. V., Hall E. J. (1951). Angiomyolipoma of the kidney. *The Journal of Urology*.

[6] Eble J. N. (1998). Angiomyolipoma of kidney. *Seminars in Diagnostic Pathology*.

[7] Daskalopoulos G., Karyotis I., Heretis I., Anezinis P., Mavromanolakis E., Delakas D. (2004). Spontaneous perirenal hemorrhage: a 10-year experience at our institution. *International Urology and Nephrology*.

[8] Lemaitre L., Claudon M., Dubrulle F., Mazeman E. (1997). Imaging of angiomyolipomas. *Seminars in Ultrasound CT and MRI*.

[9] Wang L. J., Lim K. E., Wong Y. C., Chen C. J. (1997). Giant retroperitoneal angiomyolipoma mimicking liposarcoma. *British Journal of Urology*.

[10] Mitchell T. L., Pippin J. J., Devers S. M. (2000). Incidental detection of preclinical renal tumors with electron beam computed tomography: report of 26 consecutive operated patients. *Journal of Computer Assisted Tomography*.

[11] Cheng L., Gu J., Eble J. N. (2001). Molecular genetic evidence for different clonal origin of components of human renal angiomyolipomas. *American Journal of Surgical Pathology*.

[12] Kattar M. M., Grignon D. J., Eble J. N. (1999). Chromosomal analysis of renal angiomyolipoma by comparative genomic hybridization: evidence for clonal origin. *Human Pathology*.

[13] Sepp T., Yates J. R. W., Green A. J. (1996). Loss of heterozygosity in tuberous sclerosis hamartomas. *Journal of Medical Genetics*.

[14] Martignoni G., Pea M., Rocca P. C., Bonetti F. (2003). Renal pathology in the tuberous sclerosis complex. *Pathology*.

[15] Zamboni G., Pea M., Martignoni G. (1996). Clear cell “sugar” tumor of the pancreas: a novel member of the family of lesions characterized by the presence of perivascular epithelioid cells. *American Journal of Surgical Pathology*.

[16] Silva E. G., Deavers M. T., Bodurka D. C., Malpica A. (2004). Uterine epithelioid leiomyosarcomas with clear cells: reactivity with HMB-45 and the concept of PEComa. *American Journal of Surgical Pathology*.

[17] Harabayashi T., Shinohara N., Katano H., Nonomura K., Shimizu T., Koyanagi T. (2004). Management of renal angiomyolipomas associated with tuberous sclerosis complex. *The Journal of Urology*.

[18] Pan C.-C., Yu I.-T., Yang A.-H., Chiang H. (2003). Clear cell myomelanocytic tumor of the urinary bladder. *American Journal of Surgical Pathology*.

[19] Birkhaeuser F., Ackermann C., Flueckiger T. (2004). First description of a PEComa (perivascular epithelioid cell tumor) of the colon: report of a case and review of the literature. *Diseases of the Colon & Rectum*.

[20] Yanai H., Matsuura H., Sonobe H., Shiozaki S., Kawabata K. (2003). Perivascular epithelioid cell tumor of the jejunum. *Pathology Research and Practice*.

[21] Folpe A., Goodman Z. D., Ishak K. G. (2000). Clear cell myomelanocytic tumour of the falciform ligament/ligamentum teres: a novel member of the perivascular epithelioid clear cell family of tumours with a predilection for children and young adults. *The American Journal of Surgical Pathology*.

[22] Martignoni G., Pea M., Reghellin D., Zamboni G., Bonetti F. (2007). Perivascular epithelioid cell tumor (PEComa) in the genitourinary tract. *Advances in Anatomic Pathology*.

[23] Diment J., Colecchia M. (2003). Myomelanocytic tumor of the thigh. *American Journal of Surgical Pathology*.

[24] Folpe A. L., Mckenney J. K., Li Z., Smith S. J., Weiss S. W. (2002). Clear cell myomelanocytic tumor of the thigh: report of a unique case. *The American Journal of Surgical Pathology*.

[25] Bleeker J. S., Quevedo J. F., Folpe A. L. (2012). Malignant perivascular epithelioid cell neoplasm: risk stratification and treatment strategies. *Sarcoma*.

[26] Dimmler A., Seitz G., Hohenberger W., Kirchner T., Faller G. (2003). Late pulmonary metastasis in uterine PEComa. *Journal of Clinical Pathology*.

[27] Martignoni G., Pea M., Rigaud G. (2000). Renal angiomyolipoma with epithelioid sarcomatous transformation and metastases: demonstration of the same genetic defects in the primary and metastatic lesions. *The American Journal of Surgical Pathology*.

[28] Sale G. E., Kulander B. G. (1988). ‘Benign’ clear-cell tumor (sugar tumor) of the lung with hepatic metastases ten years after resection of pulmonary primary tumor. *Archives of Pathology and Laboratory Medicine*.

